# Hypocholesterolemic Effect and *In Vitro* Pancreatic Lipase Inhibitory Activity of an *Opuntia ficus-indica* Extract

**DOI:** 10.1155/2015/837452

**Published:** 2015-05-11

**Authors:** Eduardo Padilla-Camberos, Jose Miguel Flores-Fernandez, Ofelia Fernandez-Flores, Yanet Gutierrez-Mercado, Joel Carmona-de la Luz, Fabiola Sandoval-Salas, Carlos Mendez-Carreto, Kirk Allen

**Affiliations:** ^1^Biotecnología Médica y Farmacéutica, Centro de Investigación y Asistencia en Tecnología y Diseño del Estado de Jalisco, AC, Av. Normalistas 800, Colonia Colinas de la Normal, 44270 Guadalajara, JAL, Mexico; ^2^Instituto Tecnológico Superior de Perote, Km 2.5 Carretera Perote-México Colonia Centro, 91270 Perote, VER, Mexico; ^3^Lancaster Medical School, Lancaster LA1 4YG, UK

## Abstract

Cholesterol control is fundamental for prevention of cardiovascular disorders. In this work, the hypocholesterolemic activity of an aqueous *Opuntia ficus-indica* extract (AOE) was tested in triton-induced mice. The inhibitory activity on pancreatic lipase enzyme was evaluated *in vitro* by the same extract. Furthermore, polyphenol content of the extract was evaluated. Hypercholesterolemia was induced in three groups of mice by intraperitoneal administration of Triton WR-1339. After induction of hypercholesterolemia, the groups were treated with an AOE (500 mg/kg) and saline solution and the positive control group with orlistat, respectively. Cholesterol levels were measured 24 h later in peripheral blood. The levels of blood cholesterol after administration of AOE significantly decreased compared to negative control. The inhibitory activity of AOE on pancreatic lipase enzyme was evaluated at concentrations from 60 to 1000 *μ*g/mL. The AOE inhibited the pancreatic lipase with an IC_50_ = 588.5 *μ*g/mL. The AOE had a high content of polyphenolic compounds. These results show that AOE is able to prevent hypercholesterolemia by pancreatic lipase inhibition, in part due to its polyphenolic compounds.

## 1. Introduction

Cardiovascular disease is the most common cause of death globally, including Mexico [1]. The Mexican population is prone to develop such a disease because of its high saturated fat intake. Bioactive compounds found naturally in variable concentrations in the diet can be used to improve health or reduce the risk of cardiovascular disease. The genus* Opuntia*, with about 200 species, belongs to the family Cactaceae. They are grown in several countries including Mexico [2]. It is commonly known as “Nopal” and has the potential to be used with beneficial effects on health.* Opuntia* spp. has been used traditionally to treat a variety of diseases including metabolic syndrome and diabetes [3]. Several studies of plants in this genus have revealed hypoglycemic activity [4–6].* Opuntia* can have multiple effects on metabolism, in regulating glucose, lipids, total cholesterol, high density lipoprotein, and low density lipoprotein [7]. However, there are few studies on the most common culinary species,* Opuntia ficus-indica*, and the mechanism of hypocholesterolemic activity is not clear.

*Opuntia* species, like other fruits and vegetables, are rich in polyphenols, which are the first dietary antioxidants [8]. Polyphenolic compounds have shown antioxidant, anti-inflammatory, enzyme inhibition, antimicrobial, antiallergic, antitumor, and antidiabetic activities [9–11]. Phenols inhibit the pancreatic lipase enzyme [12] which catalyze the hydrolysis of triglycerides to be absorbed by the body. By inhibiting the function of this enzyme, the cholesterol level is reduced and hyperlipidemia is prevented [13, 14]. Therefore in this study we evaluated the polyphenolic content of extracts of* Opuntia ficus-indica* obtained with different solvents. We determined the hypocholesterolemic activity in triton-induced mice. In addition, we evaluated the inhibitory activity* in vitro* of the pancreatic lipase enzyme by an aqueous* Opuntia ficus-indica* extract (AOE) to explore a possible mechanism of its hypocholesterolemic activity.

## 2. Materials and Methods 

### 2.1. Samples

Cladodes of* Opuntia ficus-indica* variety Milpa Alta were obtained from Morelos state in Mexico.

### 2.2. Chemicals Reagents and Kits

Commonly used chemicals were of reagent grade or better. MOPS (morpholinepropanesulphonic acid), p-NPB (p-nitrophenylbutyrate), Triton WR-1339, Orlistat, and Folin-Ciocalteu reagent were purchased from Sigma-Aldrich Chemical Company (St. Louis, MO, USA). QuantiChrom Lipase Assay Kit was purchased from BioAssay Systems (BioAssay Systems, Hayward, CA, USA).

### 2.3. * Opuntia ficus-indica* Extracts

Cladodes of* Opuntia ficus-indica* were dried at 37°C in a stove, pulverized with the aid of a blender, and passed through a 0.6 mm mesh sieve; then 0.5 g of dehydrated* Opuntia ficus-indica* was mixed with 10 mL of different solvents: (a) hexane-water, (b) chloroform-water, (c) methanol-water (50 : 50 v/v), and (d) water. Each mixture was allowed to stand 1 h and centrifuged at 2500 rpm for 15 min and then was stored at 4°C until further analysis.

### 2.4. Determination of Total Polyphenolic Content

Total polyphenolic content of the extracts was assessed using Folin-Ciocalteu reagent. Forty microliters of each extract was added in 750 *μ*L (0.2 M) reagent and was allowed to stand at room temperature for 5 min. A sodium bicarbonate solution (7.5%) was added to the mixture and stored at room temperature for an additional 2 h. The absorbance was measured at 765 nm by a microplate spectrophotometer (Bio-Rad Laboratories; Philadelphia, PA). The total polyphenolic contents were expressed as *μ*g of gallic acid equivalents per gram (GAE/g) of the extract [15]. The standard curve of known concentrations of gallic acid was prepared simultaneously with the test samples. All samples were assayed in triplicate.

### 2.5. Hypocholesterolemic Activity* In Vivo*

Eight-hour fasted male Balb-c mice were randomly assigned to three different groups (*n* = 6 per group). For the three groups, hypercholesterolemia was induced by administration of Triton WR-1339 (300 mg/kg body weight) by intraperitoneal administration [16]. A positive control group was treated with orlistat (50 mg/kg), a negative control group with saline solution, and the experimental group with AOE (500 mg/kg body weight). Administration was done intragastrically at a dose volume of 100 *μ*L on a single occasion immediately after hypercholesterolemia induction. Sixteen hours after administration the mice were fasted for 8 h and then cholesterol was measured. Cholesterol was measured with Accutrend Plus (Roche Diagnostics, GmbH Mannheim, Germany) portable equipment in all groups at baseline and after administration.

Animal care and management were carried out under the guidelines of Mexican Official Standard NOM-062-ZOO-1999. All mice were housed in an environment of 23 ± 2°C, 54 ± 11% relative humidity, 10–15 air changes per hour ventilation rate, and 12 : 12 hours of dark and light cycle and provided with standard diet and purified water* ad libitum*.

### 2.6. Inhibition of Pancreatic Lipase

Inhibition of pancreatic lipase was measured according to Jang et al. [17]. Thirty *μ*L of pancreatic lipase dissolved in MOPS-EDTA buffer (10 mM MOPS and 1 mM EDTA, pH 6.8) was added to 0.85 mL of TRIS buffer (100 mM Tris-HCl, 5 mM CaCl_2_, pH 7.0). Then, 100 *μ*L of aqueous extract at different concentrations (60–1000 *μ*g/mL) or orlistat (0.5–8.0 *μ*g/mL) was mixed with 20 mL of substrate solution p-NPB 10 mM and the enzymatic reaction was allowed to proceed for 15 min at 37°C. Pancreatic lipase activity was determined by measuring the hydrolysis of p-NPB to p-nitrophenol using the QuantiChrom Lipase Assay Kit according to manufacturer's instructions. The absorbances were measured at 412 nm using an ELISA reader after 10 and 20 min. Enzyme activity was calculated with the following formula:

(1) $$\begin{array}{c} \text{Activity}=\left[\frac{\left(\text{Abs}\;20\;\min-\;\text{Abs}\;10\;\min\right)}{\left(\text{Abs}\;\text{calibrator}-\text{Abs}\;\text{water}\right)}\right]*735. \end{array}$$

The IC_50_ of the test sample was obtained from probit analysis.

### 2.7. Statistical Analysis

All data were expressed as mean ± SD. The data were analyzed by one-way ANOVA followed by Tukey's post hoc test for multiple comparisons using Statgraphics 5.1 software. A *P* value < 0.05 was considered significant.

## 3. Results and Discussion

Four extracts of cladodes of* Opuntia ficus-indica* were obtained with different solvents (hexane-water, chloroform-water, and methanol-water (50 : 50 v/v)) and water. The aqueous* Opuntia ficus-indica* extract (AOE) showed the highest total polyphenolic content with 524.4 ± 49.5 *μ*g of GAE/g, followed by the methanolic extract with 434.5 ± 15.2 *μ*g of GAE/g (Table 1). The total polyphenolic content of hexane and chloroform extracts was lower. Due to high polyphenol content, the aqueous extract of* Opuntia ficus-indica* was selected for the subsequent experiments.

The active components reported in water extracts of* Opuntia ficus-indica* cladodes are phenolics acids and flavonoids like myricetin, rutin, and betalain [18]. Additionally, the presence of linoleic acid has been reported as the main fatty acid founded in* Opuntia ficus-indica* cladodes with 34.87% [19].

The polyphenol content of AOE was similar to reports of other varieties of* Opuntia ficus-indica*, such as varieties Jalpan and Villanueva with 318.1 and 593.1 *μ*g GAE/g, respectively [20], and its content is higher than some fruits such as melon (259.2 *μ*g GAE/g) and orange (358.9 *μ*g GAE/g) [21]. This suggests that* Opuntia ficus-indica *could have greater effect in lowering cholesterol, since some polyphenolic compounds inhibit the functions of the enzymes involved in the hydrolysis of carbohydrates and fats [12].

**Figure 1:**
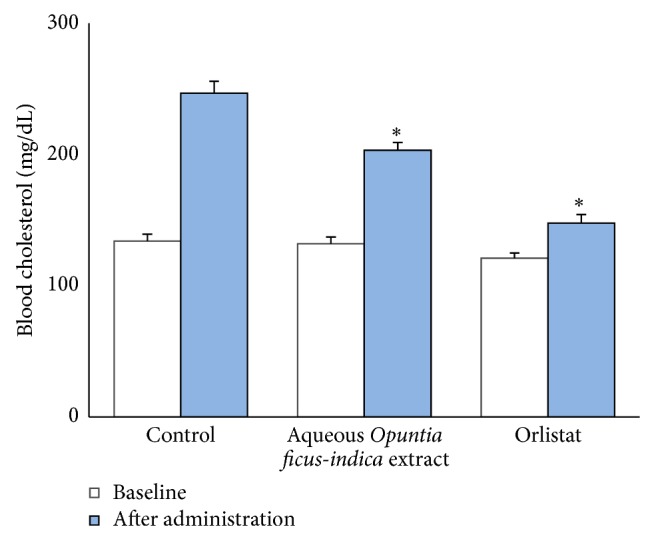
Hypocholesterolemic activity of aqueous extract of* Opuntia ficus-indica *(*n* = 6 per condition). ^∗^Significantly lower than control after administration (*P* < 0.05 by ANOVA with Tukey's post hoc test). Baseline cholesterol was not significantly different across condition.

In animal experiments, mice induced with Triton WR-1339 reached cholesterol levels of 246.5 mg/dL in comparison to the basal level of 134 mg/dL before administration. The levels of cholesterol after administration of AOE significantly decreased the cholesterol levels to 203.1 mg/dL. Positive control orlistat showed the lowest cholesterol levels with 147.8 mg/dL (*P* < 0.05, Figure 1). Previous studies have shown hypolipidemic effect of* Opuntia* water extracts in rabbits [22]. Our results are similar to extracts of* Ocimum basilicum* [16].

**Figure 2:**
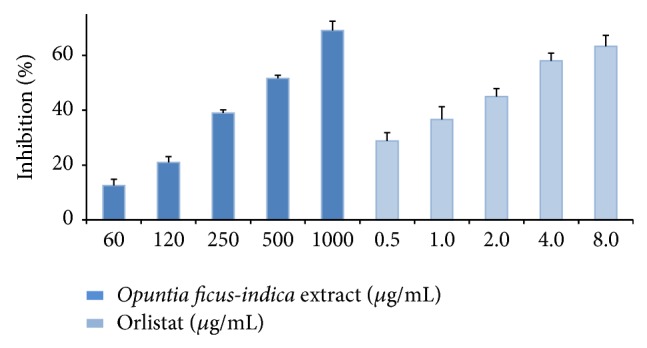
Inhibition of pancreatic lipase by aqueous extract of* Opuntia ficus-indica*. Orlistat was used as positive control.

The mechanism of hypocholesterolemic activity of* Opuntia ficus-indica* has not been described, and we hypothesize that the aqueous extract of* Opuntia ficus-indica* inhibits pancreatic lipase, for which an* in vitro* study of inhibitory activity of AOE on pancreatic lipase was carried out. The aqueous extract inhibited pancreatic lipase in dose-dependent manner. The activity* in vitro* was of 12.6, 20.9, 38.9, 51.5, and 69.1% at concentrations of 60, 120, 250, 500, and 1000 *μ*g/mL, respectively. Orlistat, a pancreatic lipase inhibitor, inhibited the enzyme activity by 28.8, 36.6, 44.9, 58, and 63.3% at concentrations of 0.5, 1.0, 2.0, 4.0, and 8.0 *μ*g/mL, respectively (Figure 2). The IC_50_ values calculated were 588.5 *μ*g/mL for AOE and 1.57 *μ*g/mL for orlistat. This result shows that the inhibition of pancreatic lipase caused by aqueous extract of* Opuntia ficus-indica* is similar to crude extracts of Korean plants such as* Rubi fructus*,* Corni fructus*,* Salicis radicis*, and* Geranium nepalense*, where their inhibition has been reported as 31–38% [23].

Pancreatic lipase is a key enzyme in dietary triacylglycerol absorption, hydrolyzing triacylglycerol to 2-monoacylglycerol and fatty acids. It is well known that dietary fat is not directly absorbed from the intestine unless it has been subjected to the action of pancreatic lipase [24]. Some natural components such as proteins and saponins inhibit gastrointestinal lipases [25], preventing the hydrolysis of dietary fat, thus reducing subsequent intestinal absorption of lipolysis products. This may explain why* Opuntia ficus-indica* decreased cholesterol levels after mice were induced with hypercholesterolemia. These results suggest that* Opuntia ficus-indica* prevents the hydrolysis of dietary fat possibly in the small intestine and reduces the subsequent intestinal absorption of dietary fat.

The polyphenolic extracts from a number of plants have been shown to be effective inhibitors of the intestinal pancreatic lipase enzyme systems [12, 15].

Our results suggest that the high polyphenolic content of AOE is in part responsible for the inhibitory activity on pancreatic lipase, but further preclinical and clinical studies are recommended.

## 4. Conclusions 

This study conducted on* Opuntia ficus-indica* confirms previously observed biological activity in other species of genre* Opuntia* and proposed an action mechanism based on enzyme inhibition. The present study is the first report that shows the fact that* Opuntia ficus-indica* aqueous extract is able to inhibit enzymatic function of pancreatic lipase which is responsible for hydrolyzing fatty acids. When these are hydrolyzed, they are absorbed by cells and this causes increase in levels of lipids.

It was demonstrated that the* Opuntia ficus-indica* extract* in vivo* lowers total cholesterol levels in the blood. Since there is overproduction of this species, it is of great importance to use the extract of* Opuntia ficus-indica* as a basis for development of a hypocholesterolemic agent or functional foods or drinks for persons with high cholesterol levels.

## Figures and Tables

**Table 1:** Total polyphenolic content.

Sample	(mg of GAE/g)
Hexane extract	106.9 ± 15.2^a^
Chloroform extract	85.4 ± 9.1^a^
Methanol extract	434.5 ± 15.2^b^
Water extract	524.4 ± 49.5^c^

Values are means ± SD of 3 determinations. Different superscripts are significantly different (*P* < 0.05).
